# Treatise on Cholera Asphyxia

**Published:** 1832-04-01

**Authors:** 


### VIII.

TREATISE ON CHOLERA ASPHYXIA; OR EPIDEMIC CHOLERA, AS IT APPEARED IN ASIA, AND MORE RECENTLY IN EUROPE, &c. By *George Hamilton Bell.* Second Edition, very greatly enlarged. Longman and Co. January, 1832.

WE are rejoiced that this excellent work has reached a second edition—and that both enlarged and improved. The fourth section, on the propagation of cholera, is handled in such a masterly manner, that we have deemed it a public benefit to our readers to print it in our EXTRA-LIMITES, in order to give it an extensive circulation. In this short article, we shall chiefly allude to the other new matter contained in the second edition, more especially as relates to the disease as it has occurred in the North of this kingdom.

When the epidemic broke out at Haddington, Dr. Meikle and Mr. Bell were deputed by the Edinburgh Board of Health to proceed, in company with Major Macdonald, to the new scene of invasion. The following was their report.

"On our arrival at this place, accompanied by Major Macdonald of the Central Board of Health, London, we found the Haddington Board of Health sitting, to which we were introduced by Mr. Riddell, Sheriff-substitute. The medical men of the town were in attendance (five*). The letter from the Secretary of the Edinburgh Board having been read, the President of the Haddington Board assured us of their willingness to co-operate with us in any inquiries we might think necessary, and we take this opportunity of observing, that this assurance was acted upon to the fullest extent.

We were informed that no new cases of Cholera had occurred since yesterday morning, and that there had been no deaths since the 28th. That six cases remained under treatment, three of which were almost well, and the others convalescent. That the medical men were convinced the disease was pestilential Cholera; but that every endeavour to trace its source had failed. At our request the medical men accompanied us to visit their patients.

1. The first place we were taken to was the suburb called the Nungate, on east side of the river Tyne. The case was that of a woman of the town, a confirmed drunkard. She had been attacked, we were informed, with symptoms of Cholera, during the night of the 28th-29th. When first seen at half-past 8 o'clock, a. m. of the 28th, she had no pulse; countenance sunk; skin cold and livid; cramps in lower extremities. Tongue white; much thirst; vomiting and purging of fluid having the appearance of meal and water. These symptoms disappeared under the use of stimulants. When we saw her (noon 30th), pulse 96, and weak; skin about natural temperature. She was *blind*, and was apparently suffering under the influence of narcotics. The blindness had just been discovered.

2. The next case we saw was that of a cabinet-maker, living in a clean and comfortable house in Haddington, on the west side of the river, opposite to the residence of the above-mentioned case, and about 100 yards in a direct line from her house, though, by the bridge, the distance must be a quarter of a mile. It was in this quarter of the town that the disease first broke out. This man is reported to have been attacked at 3 o'clock of the morning of the 29th. When first seen, half-past 8 o'clock a. m., countenance pale, skin below natural heat, but not cold; spasms in lower extremities, vomiting and purging of watery fluid; thirst, heat, and oppression at chest; pulse feeble and quick. Recovering under use of stimulants. No symptoms of Cholera when we saw him; no fever; had made water.

3. The next case to which we were taken was that of Wilson, a carter, a very powerful man, of a dissolute character, living in the centre of the town. He is reported to have been attacked with cramps in the extremities on the 28th, after having been engaged in sinking a well. When first seen, the cramps were severe, and he had pain at the pit of the stomach; neither vomiting nor purging; no collapse. Immediately bled to 24 ozs.; and opiates continued to be administered during the night. When seen by us, pulse high and tongue foul.

In all this we of course have seen nothing to authorize us to say that Cholera prevails at Haddington, on our own observation; but, from the reports of the medical men, who have treated the cases which have occurred, we have no he-

* "Drs. Black, Lorimer, Howden, Cruickshanks, and Burton."

sitation in saying, that these must have been cases of the pestilential disease at present prevailing at Newcastle. The case No. 1. is the only one of those we have seen, presenting the character of a patient recovering from Cholera; and we are of opinion, that, in this case, the woman is in great danger of a fatal state of congestion in the head.

Our next object was to examine the evidence on the question of the source of the disease.

We found that three cobblers had left Newcastle in search of work on the 14th instant, travelled on foot, and arrived in Haddington on the 19th. They had not fled from Newcastle to escape from the Cholera, but to escape starvation. For though they had heard a good deal about it, no cases had occurred near to where they lived; *they had never seen any one ill of the disease, and none of their acquaintance had suffered from it so far as they knew*. This was the statement of one of them whom we examined, and we were informed similar statements had been made by the others. *The first case, that of William Craig, occurred on the 18th, the day before these men reached Haddington. They had formerly been acquainted with this man, but did not see him after their arrival. These men live in the neighbourhood of the parts of the town where the disease has prevailed.*

The Board at Haddington have only been able to trace communication among those attacked in one instance. Two of the women, whose cases proved fatal, live in different storeys of the same house, or *land*. And it appears, that although not intimate acquaintance, they had spoken to each other on the previous day, or on the day on which the first was attacked, and that on the day following the second was seized with the disease.

All the cases have occurred within a circle of about 100 yards in diameter (but No. 3), and in a low and filthy part of the town on the banks of the river.

George Meikle.
G. Hamilton Bell.

Haddington, 30*th December*, 1831." 232.

After the minute detail of some cases treated at Haddington by Drs. Burton and Lorimer of Haddington, and Messrs. Meikle and Stevenson, surgeons, Mr. Stevenson remarks as follows:—

"With respect to the mode of propagation of this disease in Haddington, all attempts to prove its introduction from Newcastle and Sunderland have completely failed; the disease first appeared on the Haddington side of the town, close to the river Tyne; at this part the river is dammed up and stagnant. At Nungate, Mrs. Macgleish was attacked, and died. Pearson's wife was attacked on the Haddington side of the town, close to the river, and died: he then went with his children and niece, Margaret Thomson, and took up his residence in Dunbar's house, which is also near the river, in an ill-ventilated dirty lane. Here Pearson immediately took the disease, and died after an illness of only eight hours,—his niece was attacked while he was under treatment, and Dunbar also complained that evening, but, on more minute inquiry, it was found he had been indisposed all the day before. I see no proof whatever, therefore, of contagion in this case. The day after the death of Pearson, James Wingate was seized: his house is in the same lane, three houses removed from Dunbar's: all communication was denied. On the opposite side of the river (Nungate) the next case makes its appearance (Laurie) in a lane behind Mrs. Macgleish's house, it is positively stated by his wife that there was no communication whatever between the people of the two houses; in fact, Laurie had been ill of diarrhœa, and confined to his house for some days. This subject is still undergoing strict investigation on the spot, and it is to be hoped some light will be thrown on this obscure subject. As far as we are warranted in inferring from these few cases, the disease would seem to be more under the influence of locality than of contagion, especially when we see people whom terror induces to shut their doors and seclude themselves, are not exempt from that dreadful malady.

In these sentiments Mr. Meikle entirely concurs.

JAMES STEVENSON,
Surgeon, Madras Establishment.

20, EAST CUMBERLAND STREET, *5th January*, 1832." 240.

Mr. Bell received from Mr. Steele the following case, which is very interesting for several reasons. It is a decided case of cholera asphyxia, in Mr. Bell's opinion; and was exceedingly well treated by Mr. Steele, from first to last. It shews the advantage of venesection, even in the stage of collapse, and that of purgation, to ward off the fever which so generally supervenes.

" ADAM'S ROW, PARISH OF NEWTON.

*Jan.* 6, 2 p. m.—Mrs. Ross, æt. 35. Complains of severe pains in the bowels, coming on at short intervals, with contraction of the muscles at the umbilicus;—has been vomiting a fluid, resembling in its appearance barley-water, and evacuating per anum a fluid more nearly resembling perfectly pure water, with a small quantity of mucus diffused through it;—complains also of sickness and vertigo, with feeling of weight and burning heat at the præcordia;—severe spasms in the feet, legs, thighs, hips, and hands;—great prostration of strength; with thirst, and an urgent desire for cold water. Features sunk, livid, and deathlike; eyes dim and heavy; hands and feet cold; the other parts of the body not cold, but considerably below the natural warmth. The mouth inside is warm, but the breath is cool—nearly cold—respiration unaffected. Pulse 116, very small, at the wrist scarcely perceptible. Tongue white and moist. Has voided no urine since the attack came on.

For the last eight days she has been troubled occasionally with diarrhœa, accompanied with pains in the bowels,—last night, however, on going to bed, she felt perfectly well, but was awoke at four this morning with griping pains in the belly. She arose from bed at six, for the purpose of commencing her daily domestic labours, and felt a call to go to stool,--the evacuation was copious and natural, but rather loose. Before having time to dress, she became so sick, that she was under the necessity of immediately returning to bed;—her feet, legs, and hands now became cold, and affected with spasms;—she had another alvine evacuation about two hours after the first, which was dark-coloured and watery, and since that time has had constant sickness, with vomiting and purging of the peculiar watery fluid, as stated above,—the spasms at same time becoming more severe, and extending to the thighs and hips. She had, about an hour ago, an opium pill of a grain and a half, with a glass of whiskey, and at present feels rather easier.

I immediately removed about 14 ounces of blood from her arm, which was all that could be got away, and even that with much difficulty. The blood was thick and dark-coloured; and during its flow she became very sick, and vomited about 8 ounces of the whitish watery fluid before mentioned. Her pulse at the same time sunk, and could not be felt at the wrist. When the retching had subsided, she swallowed a pill, consisting of two grains of camphor and ½ grain of opium, washing it down with half a glass of brandy mixed with water,—was ordered to repeat the same every half hour,—to apply a large sinapism to the belly, with bottles filled with warm water to the feet, legs and other parts of the body.

5, p. m.—She has had no evacuations per anum since last report, and has vomited but little; what has been ejected from the stomach, however, has still the same appearance, but is imbued with something of a brownish colour, probably the brandy or dissolved opium. Has taken six pills—has had severe cramps confined to the feet, with constant sickness and thirst—feet and hands are now warm—pulse a little improved,—ordered to take one pill every hour.

9 p. m.—Three pills taken—has vomited only once since last visit, which was about two hours ago—felt a call to evacuate the bowels, but without effect—no urine—some cramps occasionally in the toes—complains much of sickness, thirst, and headachs. Lips blue—eyes sunk, and whole appearance of countenance *very cadaverous*. Pulse 116, a little stronger. Surface of body warm and clammy.

She was again bled to about nine ounces, when she became sick, as in the morning; her pulse *fell*, and the flow of blood stopped. Blood still thick and dark-coloured.

Pills and brandy to be continued.

*Jan*. 7, 10 a. m.—At one in the morning she had taken six of the camphor and opium pills as above, when they were discontinued, and one-half ounce of the mist. camphor, with five drops laudanum and a little oil of cloves given every hour. She is now improved in her appearance: has had no spasms; body of natural warmth, and covered with a clammy perspiration. Pulse 112, soft, and of natural strength. Tongue white. Has vomited none, nor has she had any alvine discharge. Took some tea and biscuit for breakfast. She is at present giving suck, but the breasts have become quite flaccid. Complains of no pain except some degree of headach, which she describes as not very severe.

Intermitt. mist. camphor.

℞. Submur. hydrarg. gr. xxx.
Ext. colocynth, gr. x.
Syrup. simp. q. s. ut ft. mass. in pil. x. dividend; quarum sumat i. omni hora.

7 p. m.—Continues to improve: countenance resuming its natural appearance, but seems a little tinged. Only three of the pills taken, and no evacuation either upwards or downwards. Voided about fourteen ounces of high-coloured urine at 11 a. m., and again a smaller quantity in the afternoon. Had beef-tea for dinner, and tea and biscuit in the evening.

*Jan*. 8, 10 a. m.—All the pills taken. Has been vomiting and purging copiously since six this morning. The fluid ejected from the stomach is tenacious, and of a dark yellowish-brown colour; that per anum is stated to have been 'black and green,' and in smell highly offensive. She complains of pains in the epigastric and umbilical regions. Countenance anxious and clammy; eyes heavy; conjunctiva of a yellowish tinge. Skin of natural warmth, dry; pulse 132, thready; tongue brown and moist; voids urine occasionally. States that on the afternoon of yesterday her milk was abundant, but that to-day it flows less freely.

8 p. m.—Has had several very dark bilious evacuations from the bowels; and twice since the morning visit, an attack of retching with the discharge of a little frothy mucus. Urine flows freely; breasts flaccid; pulse 138, thready; tongue whitish; moist. Thirst urgent. Complains of considerable pain in the epigastric and lower part of the right hypochondriac regions, where may be felt a diffused hardness, tender to the touch. She also feels it uneasy when she moves her body, and lies easiest on the left side. Appearance of countenance as in the morning. Head uneasy, but not pained. Has had hiccup twice during the afternoon, and had an attack of it at the time of visit.

*Jan*. 9, 10 a. m.—Countenance resuming its natural appearance and expression. Complains principally of weakness, thirst, and the abdominal tenderness: no appetite for food. Pulse 128, soft. Tongue whitish. Slept occasionally during the night. No vomiting. Two dark-coloured alvine evacuations. Bilious, but not feculent. Urine voided in much the same quantity as when in health, and of same appearance. Has no milk in the right breast.

8 p. m.—Was cupped this afternoon over the pained part of the belly, but with little success; not more than two ounces of blood having been got away; pain still continues; pulse 112; soft and weak; no alvine evacuation; other symptoms as in the morning.

Applicet. Emp. Lyttæ parti dolenti abdom.

Extract of letter enclosing above case.

'*Craighall*, 10*th January*, 1832.

'In reference to the above case, I may mention, that contagion seems to have had nothing to do in its origin. The residence of the patient is at least 12 miles from Haddington, with which place I have not been able to ascertain that communication of any kind had occurred. The woman is the wife of a collier; has had eight children; is cleanly in person; of temperate habits; of healthy constitution; and in so far as propriety of conduct in every respect can constitute a ground of exemption from the disease, in so far was she entitled to consider herself safe. She has had an attack of ordinary Cholera of this country every autumn for some years past.

'It is perhaps not unworthy of remark, that upon bleeding her, the blood which flowed readily at the first, became languid in its current as the flow went on, and at last stopped altogether. That she became deadly sick and vomited, and the pulse died away; in this particular, differing, so far as I have read, from most other cases of Cholera, when the pulse is said to rise with bleeding.

(Signed) Geo. Steele, *Surgeon*.'

I may observe on this point, that it was found in India, that, unless the flow of blood from the vein became more free, and its colour improved, the symptoms described by Mr. Steele very frequently followed. And there can be little doubt, had he been able to remove blood to this extent from Mrs. Ross, the first bleeding would have sufficed, and little else would have been necessary to remove the collapse. Compare this with case in text, p. 159.

Mr. Steele informs me, under date 13th January, 1832, that the blister effectually relieved the pain and symptoms of hepatic conjestion. 'She is now (he adds) nearly well, debility being her only complaint.'" 244.

With the foregoing extracts we shall take leave of Mr. Bell, requesting our readers to peruse, with great attention, his fourth section, printed in our Extra-Limites. Sober Reason is beginning to assert her sway, and we have neither doubt nor fear that truth will be established on a firm basis before the present epidemic has left our shores. The profession and the public generally are under the deepest obligations to Mr. Bell, who maintains the talent attached to his name and to his family—for we find that he is the nephew of the late John, and the present Sir Charles Bell.

